# Dexamethasone inhibits BMP7-induced osteogenic differentiation in rat dental follicle cells via the PI3K/AKT/GSK-3β/β-catenin pathway

**DOI:** 10.7150/ijms.44231

**Published:** 2020-09-21

**Authors:** Jing Tang, Mao-Feng Qing, Min Li, Zhi Gao

**Affiliations:** 1 Department of Stomatology, The Second Affiliated Hospital of Chongqing Medical University, 76 Linjiang Road, Yuzhong District, Chongqing 400010, P.R. China.; 2 Chongqing Key Laboratory of Oral Disease and Biomedical Sciences, 426 North Songshi Road, Yubei District, Chongqing 401147, P.R. China.; 3 State Key Laboratory of Oral Diseases, West China Hospital of Stomatology, Sichuan University, No. 14, Sec. 3, Renminnan Road, 610041 Chengdu, Sichuan, P.R. China.

**Keywords:** dental follicle cells, Dexamethasone, bone morphogenetic protein 7, PI3K/AKT/GSK-3β/β-catenin

## Abstract

Impacted third molars are commonly seen in teenagers and young adults and can cause considerable suffering. Preventing eruption of the third molars can reduce pain at the source. Our previous study has shown that dexamethasone (DEX) at a certain concentration can prevent the eruption of third molars without damaging alveolar bone in Sprague-Dawley (SD) rats, but the relevant molecular mechanisms need to be explored. This study aimed to explore the effects of high concentrations of DEX on osteogenic signaling pathways, including BMP/Smad and Wnt/β-catenin pathways, in rat dental follicle cells (rDFCs) and to elucidate the possible mechanisms. The results showed that BMP7 induced osteogenic differentiation by increasing the activity of ALP and the protein levels of OPN in rDFCs. DEX decreased endogenous BMP7 and phosphorylated Smad1/5/8 expression as well as BMP7-induced osteogenic differentiation. DEX also reduced the mRNA and protein levels of β-catenin by enhancing the expression of GSK-3β. In addition, regardless of DEX intervention, overexpression of BMP7 promoted the expression of β-catenin, while knockdown of BMP7 attenuated it. Further investigation revealed that overexpression of BMP7 attenuated the DEX-mediated inhibition of AKT and GSK-3β phosphorylation, but knockdown of BMP7 exerted the opposite effects. This study suggests that high concentrations of DEX may inhibit the expression of β-catenin via the PI3K/AKT/GSK-3β pathway in a manner mediated by BMP7. The findings further illustrate the possible molecular mechanisms by which DEX prevents tooth development.

## Introduction

Third molars, commonly called wisdom teeth, usually erupt between the ages of 18 and 25. During human evolution, the space for third molar eruption has become insufficient, increasing the rates of tooth impaction 1. Impacted third molars cause considerably harmful conditions in humans, such as recurrent pericoronitis, adjacent dental caries or root absorption, and temporomandibular joint disorder syndrome. Surgical extraction has been used in clinical practice for impacted third molars that may be harmful. However, due to the unique physical locations of these teeth, extraction is difficult and very traumatic and can be associated with many postoperative complications, such as bleeding, swelling, infection and dry socket 2. Prevention of third molar eruption, if possible, will therefore reduce harm to humans. Thus far, researchers have used electrosurgical energy 3, long-pulse diode lasers 4, cryogenic techniques, sclerosing agents 5, citral 6, aspirin 7 and other techniques and agents to stop tooth development.

Dexamethasone (DEX) is a glucocorticoid that has been widely used in *in vitro* culture to induce bone marrow mesenchymal stem cells (BMSCs) and dental follicle cells (DFCs) to differentiate into osteoblastic cells 8. Previous studies have reported that high concentrations of DEX can inhibit osteogenic differentiation of BMSCs 9. Our previous study 10 (in Chinese with an English abstract) showed that DEX at a certain concentration can prevent eruption of third molars without damaging alveolar bone in Sprague-Dawley (SD) rats. However, the relevant molecular mechanisms need to be explored.

Tooth eruption and root development, especially cementum development, are closely related to dental follicles. Dental follicle cells (DFCs), which are odontogenic stem cells with self-renewal ability and multidirectional differentiation potential, originate in dental follicles 11. Since 1992, when Wise et al. 12 first reported the successful culture of rat DFCs (rDFCs), relatively advanced techniques for the culture and identification of rat, human 13 and mouse 14 DFCs have been developed.

Tooth development is a continuous, long-term and complicated biological process impacted by numerous factors. Previous studies have shown that the bone morphogenic protein (BMP) 15 and Wnt 16 signaling pathways not only participate in the interaction between epithelial cells and mesenchymal cells during the process of tooth development but also regulate osteoblast/cementogenic differentiation in rDFCs.

This study aimed to investigate the effects of high concentrations of DEX on osteogenic signaling pathways, such as the BMP/Smad and Wnt/β-catenin pathways, in rDFCs and to elucidate the possible mechanisms. The findings may help illustrate the molecular mechanisms by which DEX prevents tooth development.

## Materials and Methods

### Primary rDFC isolation, culture and identification

The procedures in the present study were conducted in accordance with the National Institutes of Health guidelines on the ethical use of animals 17. All experiments were performed with the approval of the Ethics Committee of Chongqing Medical University. The experimental rats were purchased from the Experimental Animal Center of Chongqing Medical University (License No: SYXK [Yu] 20040001).

The animal experiments were performed in biosafety cabinets. Seven-day-old SD rats were sacrificed by cervical dislocation and bathed in 75% ethanol for 10 min. Their mandibles were removed surgically and placed in phosphate-buffered saline (PBS, TBD, Tianjin, China). Then, the dental follicles were dissected from the first and second molars under a dissecting microscope. The isolated tissues were minced into 1 mm^3^ pieces with microsurgical tweezers and collected in low-glucose Dulbecco's modified Eagle medium (DMEM, Sigma, St. Louis, MO, USA) at room temperature during dissection. After being washed one time with penicillin/streptomycin solution (HyClone, Logan, UT, USA), the dental follicles were incubated in a collagenase type I (0.1-5 mg/mL, Sigma) solution for 30 min at 37 °C to partially dissociate the DFCs. Then, the samples were centrifuged for 3 min at 1,000 × *g* and incubated in a 0.25% trypsin (HyClone) solution for 3 min at 37 °C. After centrifugation for 3 min at 1,000 × *g*, the tissues were plated in Φ60 mm plastic culture dishes. The rat dental follicle tissues and rDFCs were incubated in low-glucose DMEM containing 10% fetal bovine serum (FBS, Excell, Shanghai, China), 100 U/mL penicillin, and 100 μg/mL streptomycin. The dishes were placed in a 37 °C incubator under 5% CO_2_. The growth medium was changed every 2 days. The rDFCs were passaged at a ratio of 1:2 when they reached approximately 75-80% confluence. Trypsin (0.25%) was used for differential digestion to remove the few epithelial cells that were mingled with primary rDFCs, as it digested rDFCs faster than epithelial cells. The trypsin digestion was immediately terminated with DMEM containing 10% FBS once the spindle cells were observed to contract and become round and the polygonal cells exhibited no obvious changes under a phase-contrast microscope. The mingled epithelial cells were maintained in plastic dishes, and the rDFCs were transferred with DMEM to new dishes. After 2 passages, almost no epithelial cells remained; thus, the rDFCs were purified. The cells were identified as reported previously in detail 18.

Cells at the 3^rd^ passage were used for subsequent experiments. DEX (D4902-100MG) and dimethylsulfoxide (DMSO, D2650-100ML) were purchased from Sigma-Aldrich.

### Recombinant adenovirus construction and transfection

Recombinant adenoviruses were generated with an Ad-Easy system as reported previously in detail 19,20. The coding regions of green fluorescent protein (GFP), BMP7 and red fluorescent protein (RFP) were amplified with PCR and then cloned into adenoviral shuttle vectors respectively, so did the BMP7 siRNA oligo fragments. The shuttle vectors were recombined in BJ5183 cells and transinfected into HEK293 cells to generate the corresponding recombinant adenoviruses Ad-GFP, Ad-BMP7, Ad-RFP and Ad-siBMP7 21-23. Ad-GFP was used as a control for Ad-BMP7, and Ad-RFP was used as a control for Ad-siBMP7.

Third-passage rDFCs were seeded into 6-well plates at a density of 2.5×10^5^ cells per well. Six hours later, the cells were infected with the appropriate recombinant adenovirus in the presence of polybrene (Solarbio, Beijing, China). The concentration of polybrene used in our experiments was 8 μg/mL. Fluorescence was observed and imaged under a microscope after 24 h or 48 h.

### Alkaline phosphatase (ALP) activity assay

Third-passage rDFCs were seeded on 24-well plates at a density of 5×10^4^ cells per well and treated with the appropriate recombinant adenovirus or reagents according to the experimental design. ALP activity was measured on day 5 and/or day 7. At the planned time points (when the cells were treated with adenovirus or reagents and cultured for 5 and/or 7 days), the cells in 24-well plates were fixed with 0.05% (v/v) glutaraldehyde (Sigma) at room temperature for 10 min. Then, the cells were washed 3 times with PBS and stained with a BCIP/NBT ALP chromogenic assay kit (Beyotime, Haimen, China). Once staining was complete, the plates were placed under a microscope and imaged.

### Quantitative real-time PCR

Third-passage rDFCs were seeded on 6-well plates at a density of 2.5×10^5^ cells per well and treated with the appropriate recombinant adenovirus or reagents according to the experimental design. After 24 or 48 h, at the scheduled time points, the cells were washed 3 times with PBS. Total RNA was prepared from the rDFCs with TRIzol reagent according to the manufacturer's recommendations (Invitrogen, Carlsbad, CA, USA). The concentration and purity of the RNA were measured at 260 nm and 280 nm by a micro-ultraviolet/visible light spectrophotometer, and the total RNA concentration of each group was diluted to 1 μg/μL. A volume of 1 μL of the diluted total RNA solution was used for reverse transcription (RT) in a total reaction volume of 20.0 μL. The initial reaction mixture also contained 1 µL of oligo (dt) primers and 10.0 μL of nuclease-free water, and RT was carried out according to the instructions of an RT kit (Thermo Fisher Scientific, Inc., Waltham, MA, USA). After the mixture was heated on a gradient PCR instrument for 5 min at 65 °C, 4 μL of 5× reaction buffer, 1 μL of RiboLock ribonuclease inhibitor (20 U), 2 μL of a 10 mM nucleotide mixture, and 1 μL of RevertAid reverse transcriptase (200 U/μL) were added. The RNA was reverse transcribed into cDNA at 42 °C for 60 min and at 70 °C for 5 min. Then, 1 μL of cDNA template was mixed with 1 μL each of forward and reverse primers (10 μM), 10 μL of RevertAid reverse transcriptase (200 U/μL) and 7 μL of sterilized double-distilled water. The mixture was reacted in an automatic thermal cycler (StepOnePlus, Thermo Fisher Scientific, Inc., Waltham, MA, USA). The amplification conditions were as follows: denaturation at 95°C for 3 min, annealing at 60°C for 30 s, and extension at 95°C for 1 min for 40 cycles. The expression levels of β-catenin were thus determined by real-time PCR. GAPDH was used as an internal reference gene, while β-catenin was the target gene. The sequences of the related primers were as follows: GAPDH, 5'-AAGTTCAACGGCACAGTCAAGG-3' (forward) and 5'-ACGCCAGTAGACTCCACGACAT-3' (reverse); β-catenin, 5'-GGTGAAAATGCTTGGGTCGC-3' (forward) and 5'-AGATCTGAAGGCAGTCTGTCGTAA-3' (reverse). The reaction was run in triplicate for each sample, and the mean Ct value was used for analysis. The 2^-ΔΔCt^ method was used to determine the relative gene expression level, and the data are presented as the means ± standard deviations.

### Western blotting

Third-passage rDFCs were seeded on 6-well plates at a density of 2.5×10^5^ cells per well and treated with the appropriate recombinant adenovirus or reagents according to the experimental design. At the planned time points, rDFCs were washed 3 times with PBS, lysed with RIPA buffer on ice for 15 min, and then centrifuged at 12,000 r/min at 4 °C for 4 min. Next, loading buffer was added to the lysates, and the mixtures were boiled for 5 min. The total amount of protein in each lane was 25 μg. The samples were separated by sodium dodecyl sulfate-polyacrylamide gel electrophoresis (SDS-PAGE) and transferred to polyvinylidene difluoride (PVDF) membranes. The membranes were blocked in a solution of 5% bovine serum albumin (BSA) dissolved in Tris-buffered saline with Tween 20 (TBST) for 1 h. Primary antibodies were diluted 1/1,000-1/2,000 with 5% BSA according to the manufacturer's recommendations. After blocking, the PVDF membranes were washed with TBST, incubated with the diluted primary antibodies at 4 °C overnight with slow shaking, and washed 5 more times with TBST. The corresponding secondary antibodies were diluted at a ratio of 1/1,000, and the PVDF membranes were then incubated with the diluted secondary antibodies for specific binding at 25 °C for 1 h. The membranes were washed 5 times with TBST. Finally, the target bands were visualized with ECL Western Blotting Substrate (Solarbio, Beijing, China). The results were repeated in at least three independent experiments.

### Quantitative analysis using NIH ImageJ

The results of western blot analysis and ALP staining were quantified with ImageJ software. For western blot quantification, the values of the bands were measured, and then the ratios of the target protein values to the corresponding GAPDH values were calculated and normalized for further processing. For ALP quantification, the optical densities of the images were measured and normalized. The results were repeated in at least three independent experiments.

### Statistical analysis

Statistical analysis was performed with GraphPad Prism (version 7, GraphPad Software, Inc., LA, CA, USA). One-way ANOVA was used with the Bonferroni post-test (for comparison of all groups) or the Dunnett posttest (for comparison of all groups to a control group). The data are presented as the mean ± standard deviation for each group. *P*-values less than 0.05 were considered to indicate statistical significance. All experiments were repeated at least three times.

## Results

### Effects of BMP7 on osteogenic differentiation in rDFCs

**Figure 1:**
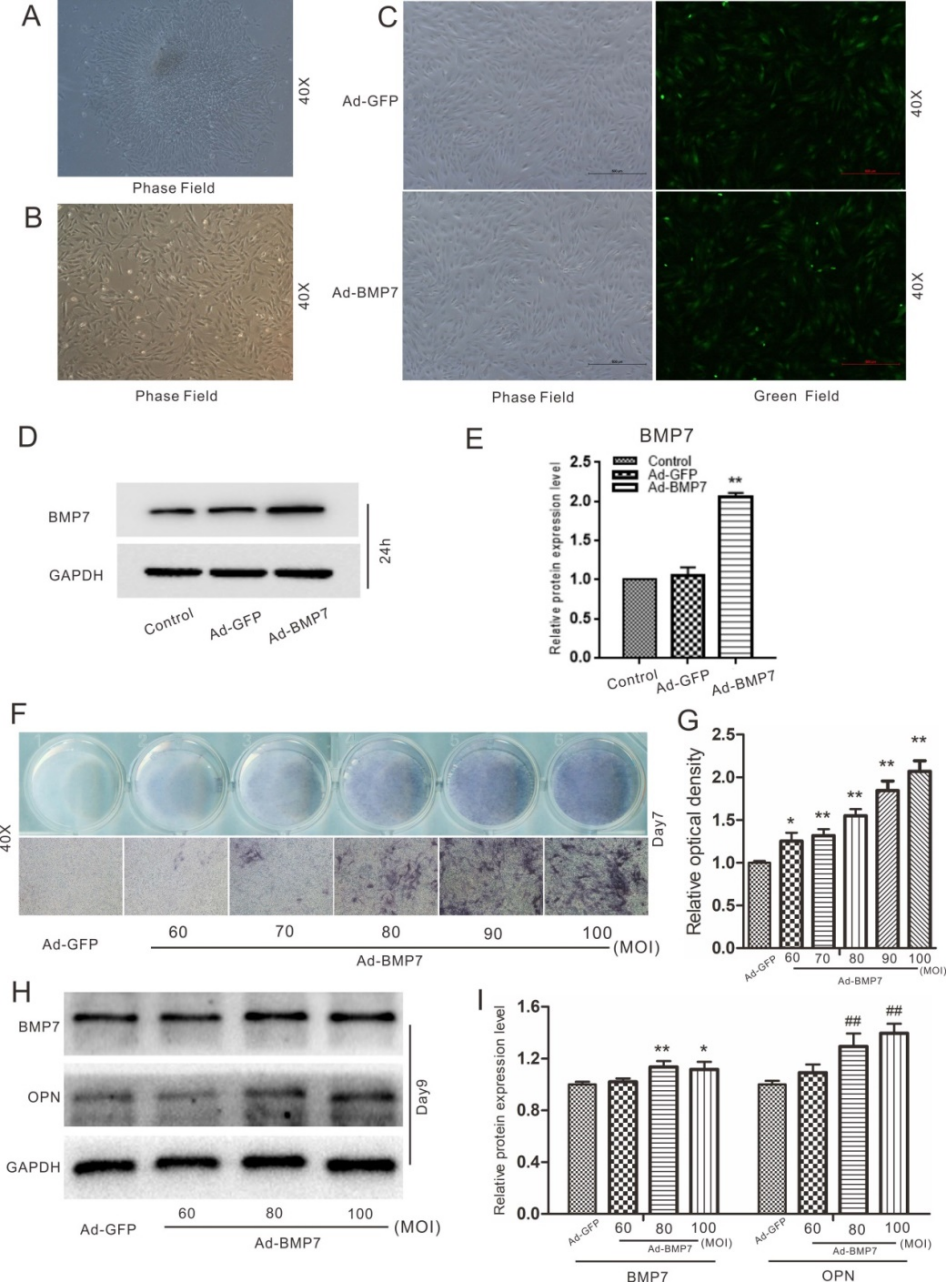
** Effects of BMP7 on osteogenic differentiation in rDFCs.** (**A**) Microscopic view of primary rat dental follicle cells (rDFCs) isolated from SD rats. (**B**) Microscopic view of 3rd passage of rDFCs. (**C**) Adenovirus mediated Ad-GFP and Ad-BMP7 in rDFCs after 24 h. (**D**) Western blot results showed the functional validation of Ad-BMP7 in rDFCs (24 h). (**E**) Quantification results of Western blot showed the functional validation of Ad-BMP7 in rDFCs. **p < 0.01 vs control. (**F**) ALP staining results demonstrated the effect of different MOI value of Ad-BMP7 on ALP activities in rDFCs, Ad-GFP was used as control. MOI, Multiplicity of Infection, means the ratio of the virus to the number of cells at the time of infection. (**G**) Quantification results of ALP staining showed the effect of different infection rate of Ad-BMP7 on ALP activities in rDFCs, *p < 0.05 vs Ad-GFP, **p < 0.01 vs Ad-GFP. (**H**) Western blot results showed the effect of Ad-BMP7 with different MOI value on the expression of BMP7 and OPN in rDFCs (Day9). (**I**) Quantification results of Western blot showed the effect of Ad-BMP7 on the expression of BMP7 and OPN in rDFCs. *p < 0.05, **p < 0.01 vs corresponding Ad-GFP; ^##^ *p* < 0.01 vs corresponding Ad-GFP.

The cells isolated from the dental follicles were pleomorphic. There were two main types of rDFCs: cuboidal/polygonal rDFCs and elongated, spindle-shaped, fibroblast-like rDFCs (Fig. 1A). The cells reached approximately 70%-80% confluency after approximately 6 days. A few epithelial cells were mingled with the primary rDFCs. The mingled epithelial cells were removed by differential digestion with 0.25% trypsin, and the rDFCs were purified at the 3rd passage (Fig. 1B). The purified rDFCs were then transfected effectively with Ad-GFP or Ad-BMP7 (Fig. 1C). The functional validation of Ad-BMP7 showed that Ad-BMP7 increased the protein expression of BMP7 after 24 h (Fig. 1D and E). The qualitative and quantitative ALP staining results showed that BMP7 overexpression increased ALP activity in an infection rate-dependent manner (Fig. 1F and G). The qualitative and quantitative western blot results showed that BMP7 overexpression also increased the protein level of the late osteogenic marker OPN (Fig. 1H and I).

### Effects of DEX and overexpression of BMP7 on endogenous BMP/Smad signaling in rDFCs

**Figure 2:**
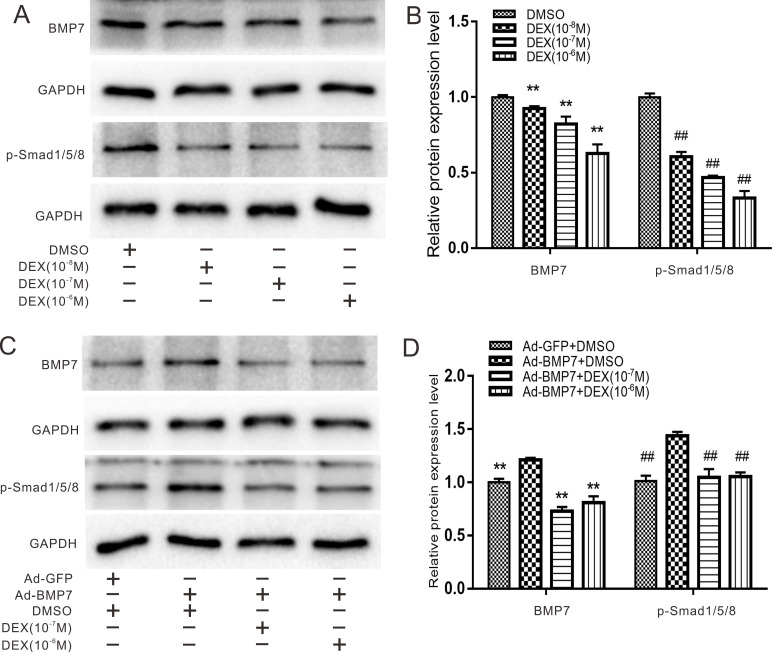
** Effects of Dex on endogenous and overexpression of BMP/Smad in rDFCs.** (**A**) Western blot results showed the effect of different concentrations of DEX on endogenous expression of BMP7 and p-Smad1/5/8 in rDFCs. (**B**) Quantification results of Western blot showed the effect of different concentrations of DEX on the endogenous expression of BMP7 and p-Smad1/5/8 in rDFCs, **p < 0.01 vs corresponding DMSO; ^##^*p* < 0.01 vs corresponding DMSO. (**C**) Western blot results showed the effect of different concentrations of DEX on the expression of BMP7 and p-Smad1/5/8 induced by BMP7 in rDFCs. (**D**) Quantification results of Western blot showed the effect of different concentrations of DEX on the expression of BMP7 and p-Smad1/5/8 induced by BMP7 in rDFCs, **p < 0.01 vs corresponding Ad-BMP7 + DMSO; ^##^*p* < 0.01 vs corresponding Ad-BMP7+DMSO.

It has been widely reported that DEX can inhibit osteogenic differentiation of stem cells via the BMP/Smad or Wnt/β-catenin pathways, so we first detected the effects of DEX on endogenous BMP7 and phosphorylated Smad1/5/8. rDFCs were treated with DEX at concentrations of 10^-8^ to 10^-6^ M for 48 h, and the qualitative and quantitative western blot results showed that DEX decreased the expression of BMP7 and phosphorylated (p-) Smad1/5/8 in a concentration-dependent manner (Fig. 2A and B). For further validation, we combined BMP7 overexpression with treatment with different concentrations of DEX (10^-7^ and 10^-6^ M) and found that DEX still reduced BMP7 and p-Smad1/5/8 expression (Fig. 2C and D).

### Effects of DEX on BMP7-induced osteogenic differentiation in rDFCs

**Figure 3:**
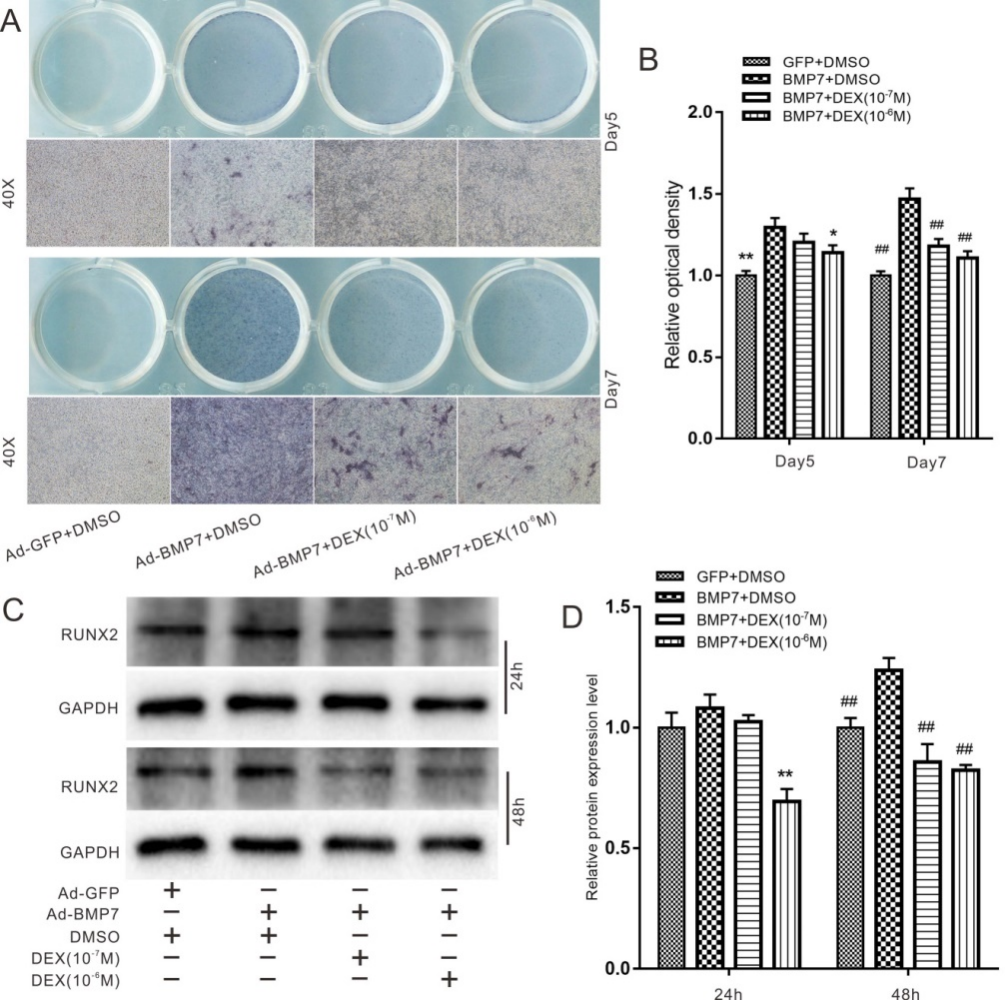
** Effects of Dex on BMP7-induced osteogenic differentiation in rDFCs.** (**A**) ALP staining results showed the effect of different concentrations of DEX on BMP7-induced ALP activities in rDFCs, Ad-GFP+DMSO was used as control. (**B**) Quantification results of ALP staining showed the effect of different concentrations of DEX on BMP7-induced ALP activities in rDFCs, *p < 0.05, **p < 0.01 vs Ad-BMP7 + DMSO (Day5); ^##^*p* < 0.01 vs Ad-BMP7 + DMSO (Day7), Ad-GFP + DMSO was used as control. (**C**) Western blot results showed the effect of different concentrations of DEX on BMP7-induced expression of RUNX2 in rDFCs, Ad-GFP + DMSO was used as control. (**D**) Quantification results of Western blot showed the effect of different concentrations of DEX on BMP7-induced expression of RUNX2 in rDFCs, **p < 0.01 vs Ad-BMP7 + DMSO (24 h); ^##^*p* < 0.01 vs Ad-BMP7 + DMSO (48 h), Ad-GFP + DMSO was used as control.

Next, we explored the effect of DEX on BMP7-induced osteogenic differentiation in rDFCs. In an ALP staining assay, we found that high concentrations of DEX (10^-7^ and 10^-6^ M) attenuated the BMP7-mediated induction of ALP activity in rDFCs (Fig. 3A). In addition, ALP activity was more obvious on day 7 than on day 5. These results were confirmed by quantitative analysis (Fig. 3B). We further used a western blot assay to determine the protein levels of the early osteogenic marker RUNX2. The results showed that high concentrations of DEX attenuated the BMP7-mediated induction of RUNX2 expression (Fig. 3C and D).

### Effects of DEX on Wnt/β-catenin in rDFCs

**Figure 4:**
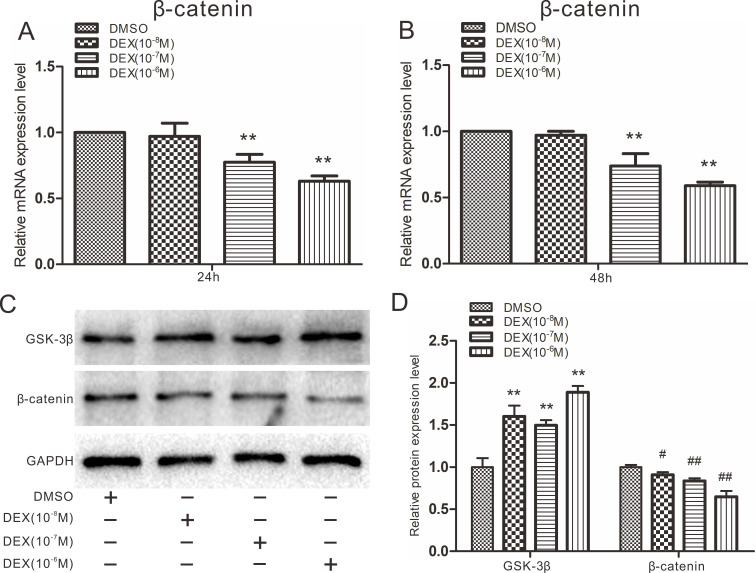
** Effects of Dex on β-catenin in rDFCs.** (**A**) Quantitative real-time PCR results showed the effect of different concentrations of DEX on endogenous expression of β-catenin in rDFCs after 24h, ** *p <* 0.01 vs DMSO. (**B**) Quantitative real-time PCR results showed the effect of different concentrations of DEX on expression of β-catenin in rDFCs after 48h, **p < 0.01 vs DMSO. (**C**) Western blot results showed the effect of different concentrations of DEX on the expression of GSK-3β and β-catenin in rDFCs. (**D**) Quantification of Western blot showed the effect of different concentrations of DEX on the expression of GSK-3β and β-catenin in rDFCs, **p < 0.01 vs corresponding DMSO; ^#^*p <* 0.05 vs corresponding DMSO, ^##^*p <* 0.01 vs corresponding DMSO.

High doses of DEX are known to block the expression of β-catenin during osteogenesis, leading to osteoporosis, so we next explored whether DEX could affect Wnt/β-catenin in rDFCs. Cells were treated with 10^-8^ to 10^-6^ M DEX. Quantitative real-time PCR results showed that 10^-7^ and 10^-6^ M DEX inhibited the expression of β-catenin in rDFCs (Fig. 4A and B). However, β-catenin expression was not significantly different between the 10^-8^ M DEX-treated group and the DMSO group. The results of a western blot assay also showed that a high concentration of DEX inhibited the protein expression of β-catenin. In contrast, the expression of GSK-3β was slightly elevated by DEX (Fig. 4C and D).

### Effects of BMP7 on β-catenin in rDFCs

**Figure 5:**
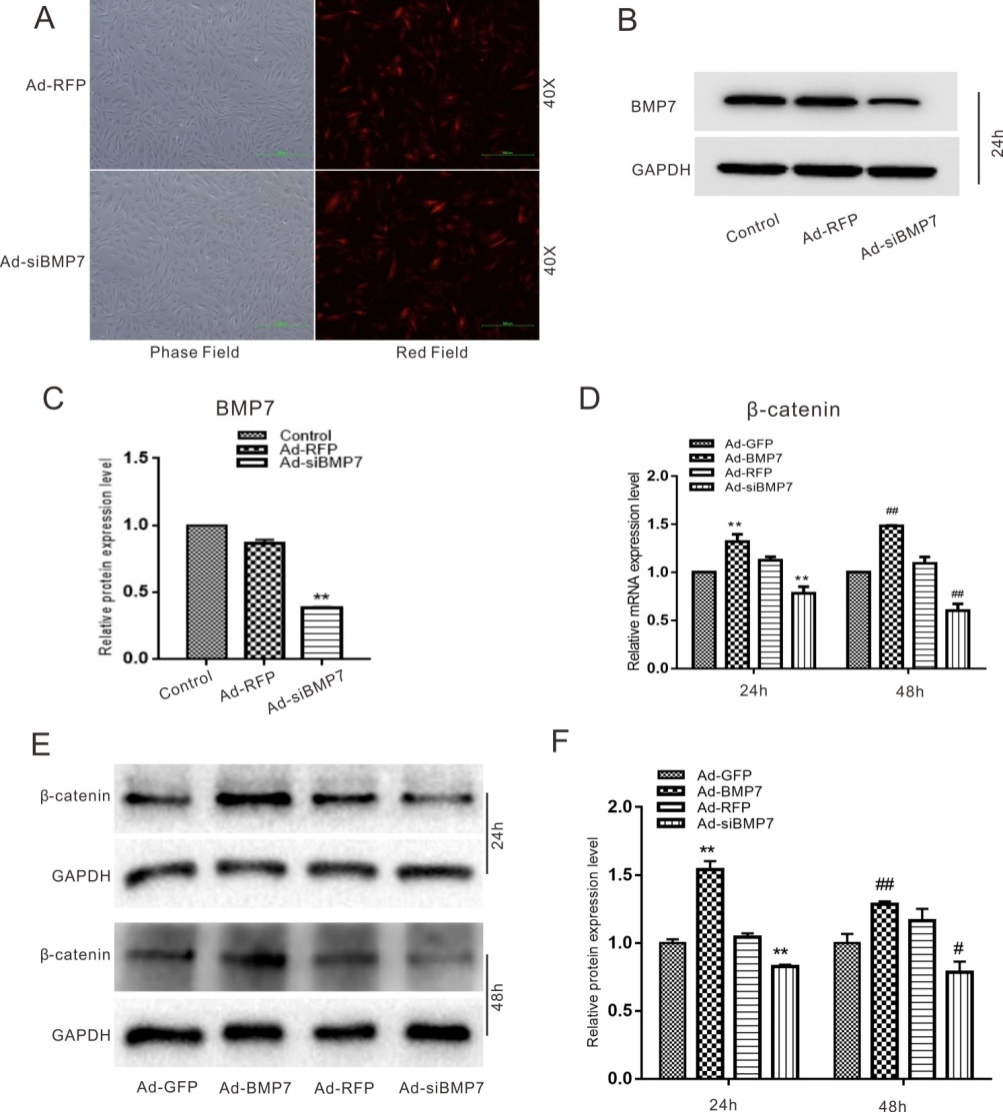
** Effects of BMP7 on the expression of β-catenin in rDFCs.** (**A**) Adenovirus mediated Ad-RFP and Ad-siBMP7 in rDFCs after 24 h. (**B**) Western blot results showed the functional validation of Ad-siBMP7 in rDFCs (24 h). (**C**) Quantification results of Western blot showed the functional validation of Ad-siBMP7 in rDFCs. **p < 0.01 vs control. (**D**) Quantitative Real-time PCR results showed the effect of BMP7 on the expression of β-catenin in rDFCs, **p < 0.01 vs Ad-GFP (24 h); ^##^*p <* 0.01 vs Ad-GFP (48 h), Ad-GFP was used as control. (**E**) Western blot results showed the effect of BMP7 on the expression of β-catenin in rDFCs. (**F**) Quantification results of Western blot showed the effect of BMP7 on the expression of β-catenin in rDFCs. **p < 0.01 vs Ad-GFP (24h); ^#^*p <* 0.05 vs Ad-GFP, ^##^*p <* 0.01 vs Ad-GFP (48 h), Ad-GFP was control.

In the following experiments, we aimed to investigate the relationship between BMP7 and β-catenin in rDFCs. Cells were transfected effectively with Ad-RFP, Ad-siBMP7 (Fig. 5A), Ad-GFP or Ad-BMP7. The functional validation of Ad-siBMP7 showed that Ad-siBMP7 decreased the protein expression of BMP7 after 24h (Fig. 5B and C). Our quantitative real-time PCR results had revealed that the mRNA expression of β-catenin was increased by overexpression of BMP7. However, the mRNA level of β-catenin was lower in the Ad-siBMP7 group (with BMP7 knockdown) than in the Ad-GFP group (Fig. 5D). The same effects were observed by means of western blotting and corresponding quantification (Fig. 5E and F).

### Effects of BMP7 and DEX on β-catenin in rDFCs

**Figure 6:**
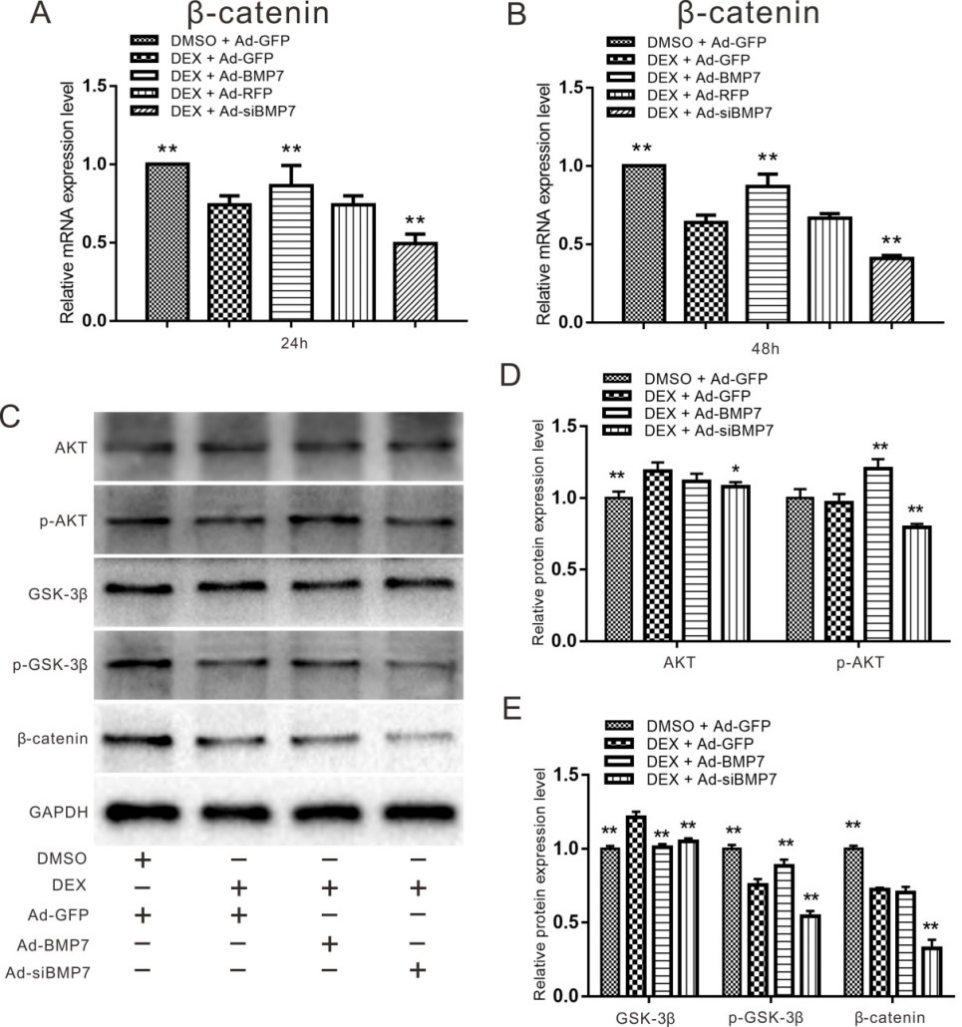
** Effects of DEX and BMP7 on β-catenin in rDFCs.** (**A**) Quantitative Real-time PCR results showed the effect of BMP7 on the expression of β-catenin inhibited by DEX in rDFCs after 24h, **p < 0.01 vs DEX + Ad-GFP, DMSO + Ad-GFP as control. (**B**) Quantitative Real-time PCR results showed the effect of BMP7 on the expression of β-catenin inhibited by DEX in rDFCs after 48h, **p < 0.01 vs DEX + Ad-GFP, DMSO + Ad-GFP as control. (**C**) Western blot results showed the effect of BMP7 on the expression of AKT, p-AKT, GSK-3β, p-GSK-3β and β-catenin mediated by DEX in rDFCs. (**D and E**) Quantification results of Western blot showed the effect of BMP7 on the expression of AKT, p-AKT, GSK-3β, p-GSK-3β and β-catenin mediated by DEX in rDFCs, *p < 0.05, **p < 0.01 vs corresponding DEX + Ad-GFP, DMSO + Ad-GFP as control.

For the following experiments, we combined DEX with Ad-BMP7 or Ad-siBMP7 to investigate whether the effect of DEX on β-catenin could be regulated by BMP7. Considering that the concentration of 10^-6^ M DEX was effective in previous experiments, we chose it for further experiments. Quantitative real-time PCR showed that the mRNA expression of β-catenin, which was inhibited by DEX, was upregulated by Ad-BMP7 after 48 h, although there was no statistical significance after 24 h (Fig. 6A and B). Conversely, knockdown of BMP7 exacerbated the DEX-mediated inhibition of β-catenin expression, indicating that BMP7 is involved in the inhibitory effect of DEX on β-catenin.

Since PI3K/AKT can be regulated by BMP signaling, we detected the expression of AKT, p-AKT, GSK-3β, p-GSK-3β and β-catenin using western blot assay. Treatment with the recombinant adenovirus and/or DEX for 48 h promoted the phosphorylation of AKT and GSK-3β, while treatment with Ad-siBMP7 inhibited the phosphorylation of AKT and GSK-3β induced by DEX. The expression of β-catenin was obviously decreased by DEX treatment combined with Ad-siBMP7 transfection (Fig. 6C). These results were confirmed by quantitative analysis (Fig. 6D and E).

## Discussion

Dental follicles play essential roles in root development and tooth eruption, and BMP 15 and Wnt 16 signaling pathways have important influences on osteoblast/cementogenic differentiation in rDFCs.

BMPs, which are members of the transforming growth factor-β (TGF-β) superfamily 24, bind to two different serine/threonine kinase receptors and mediate their signals through Smad-dependent and Smad-independent pathways. Receptor regulated-Smad (R-Smad) proteins specific to BMP pathways interact with various proteins, including the transcription factor RUNX2, to transmit specific signals in target cells. BMP signaling pathways participate in the formation and development of almost all mammalian body tissues and organs, including nerves, gastrointestinal tissues, cardiovascular tissues and teeth. In addition, BMP pathways regulate the differentiation of osteoblasts and chondroblasts, induce the formation of ectopic bone, promote the healing of fractures, and play key roles in bone development and stem cell differentiation 25. It has been demonstrated that BMP2, BMP3, BMP4, BMP5, BMP6, BMP7 and BMP9 participate in tooth development.

BMP7, a member of the BMP family, is expressed throughout the whole process of tooth development in rats and is particularly important during root development 26. Studies have shown that BMP7 promotes the differentiation and mineralization of osteoblasts by inducing PCPE1 and BMP1 to affect type I collagen 27. In previous research, fibroblasts transfected with the bmp7 gene have been implanted into bone defects to promote cartilage formation, ossification and cementum formation 28. In addition, it has been reported that mice with embryonic deletion of the BMP7 gene lack all maxillary incisors and exhibit malformed, underdeveloped or missing mandibular incisors as well as delays in upper and lower first molar development or misplaced/missing upper and lower first molars 29. These findings demonstrate that BMP7 plays an important role in the formation of cementum.

In our study, we first explored the effects of BMP7 on osteogenic differentiation in rDFCs. Through detection of the early osteogenic marker ALP and the late osteogenic indicator OPN, we proved that BMP7 induced osteogenic differentiation in rDFCs (Fig. 1). We added DEX in the following experiments and explored the effects of treatment with different concentrations of DEX and overexpression of BMP7 on endogenous BMP/Smad in rDFCs. We discovered that a high concentration of DEX decreased the expression of endogenous BMP7 and p-Smad1/5/8 (Fig. 2). Next, we explored the effects of DEX on BMP7-induced osteogenic differentiation in rDFCs. Detection of the expression of RUNX2 and ALP revealed that a high concentration of DEX inhibited BMP7-induced osteogenic differentiation in rDFCs (Fig. 3).

Wnt signaling pathways are also critical, as these pathways regulate cell proliferation, differentiation, apoptosis and intercellular interactions, playing important roles in embryonic formation, tissue repair and other biological processes 30. Previously, proteomics results have shown that Wnt signaling pathways also participate in osteoblast/cementogenic differentiation of human DFCs 31.

Wnt signaling pathways can be divided into canonical Wnt/β-catenin signaling pathways and noncanonical Wnt/Ca^2+^, Wnt/PCP, Wnt/RAPI and other pathways according to their transduction modes. The canonical Wnt/β-catenin pathways include mainly Wnt proteins, the receptor Fz, the coreceptor LRP5/6, the conduction factor β-catenin, the APC protein, glycogen synthesis kinase-3 proteins, the nuclear transcription factor TCF/LEF, downstream target genes and other extracellular factors. In canonical Wnt signaling pathways, Wnt ligands bind to the receptors Fz and LRP5/6 and activate intracellular Dishevelled (Dsh/Dvl). DVL recruits Axin and GSK-3β; destroys the complex composed of β-catenin, GSK-3β, Axin and APC; and inhibits β-catenin phosphorylation. β-Catenin accumulates in the cytoplasm, enters the cell nucleus and binds to TCF/LEF, forming a transcription complex to activate target gene expression and then regulate the processes of cell proliferation, differentiation and apoptosis 32. The effect of DEX on Wnt pathways can be investigated by detecting the expression of the key factor β-catenin. Therefore, we explored the effects of DEX and BMP7 on β-catenin in rDFCs. We discovered that DEX inhibited the expression of β-catenin by enhancing the expression of GSK-3β (Fig. 4). In contrast, overexpression of BMP7 promoted the expression of β-catenin, while knockdown of BMP7 attenuated it (Fig. 5). The following experiment explored the effect of DEX combined with BMP7 or siBMP7 on β-catenin in rDFCs. The results showed that overexpression of BMP7 upregulated the expression of β-catenin, which was inhibited by DEX, while knockdown of BMP7 exacerbated the DEX-mediated inhibition (Fig. 6A and B).

The PI3K/AKT/Gsk-3β signaling pathway is an important signaling pathway in cells that participates in the regulation of many important biological processes. Specifically, it plays a key role in inhibiting apoptosis and promoting cell proliferation by affecting the activation statuses of various downstream effector molecules 33. Since PI3K/AKT can be regulated by BMP signaling, we detected the expression of AKT, p-AKT, GSK-3β, p-GSK-3β and β-catenin (Fig. 6C, D and E). The results showed that under treatment with high concentrations of DEX, overexpression of BMP7 upregulated the expression of p-Akt and p-GSK-3β, while knockdown of BMP7 had the opposite effect. These findings reveal that DEX can regulate the expression of β-catenin and that the mechanism may be associated with BMP7 via the PI3K/AKT/GSK-3β pathway. Previous reports have demonstrated that when AKT, the downstream factor of PI3K, is phosphorylated and activated, it can deactivate GSK-3β by phosphorylating it. P-GSK-3β cannot bind with β-catenin, leading to the accumulation of high levels of β-catenin in the cytoplasm, after which β-catenin enters the cell nucleus and induces target gene transcription 34. Based on the results of our study, high concentrations of DEX may inhibit the expression and osteogenic ability of BMP7, inhibit the phosphorylation of AKT, decrease the expression of p-GSK-3β, promote the binding of GSK-3β and β-catenin, and inhibit WNT/β-catenin pathway signaling.

In conclusion, our experiments suggest that overexpression of BMP7 upregulates the expression of β-catenin, which is inhibited by high concentrations of DEX, but that knockdown of BMP7 exerts the opposite effect. We speculate that high concentrations of DEX may inhibit the expression of β-catenin via the PI3K/AKT/GSK-3β pathway in a manner mediated by BMP7. However, the exact mechanisms need further exploration. Our findings may contribute to the development of a new strategy for preventing the eruption of third molars.
